# C4orf47 contributes to the dormancy of pancreatic cancer under hypoxic conditions

**DOI:** 10.7150/jca.78993

**Published:** 2023-01-09

**Authors:** Shinjiro Nagao, Hideya Onishi, Makoto Kawamoto, Shogo Masuda, Lin Na, Shinji Morisaki, Naoya Iwamoto, Yutaka Yamada, Satoko Koga, Shu Ichimiya, Kazunori Nakayama, Akira Imaizumi, Kinichi Nakashima, Yoshinao Oda, Masafumi Nakamura

**Affiliations:** 1 Department of Cancer Therapy and Research, Graduate School of Medical Sciences, Kyushu University, Fukuoka, Japan.; 2 Department of Anatomic Pathology, Graduate School of Medical Sciences, Kyushu University, Fukuoka, Japan.; 3 Department of Stem Cell Biology and Medicine, Graduate School of Medical Sciences, Kyushu University, Fukuoka, Japan.; 4 Department of Surgery and Oncology, Graduate School of Medical Sciences, Kyushu University, Fukuoka, Japan.

**Keywords:** dormancy, pancreatic cancer, hypoxia

## Abstract

In our comprehensive analysis of pancreatic cancer pathology, we found that the C4orf47 molecule was upregulated in hypoxic environments. C4orf47 is reported to be a centrosome-associated protein, but its biological significance in cancer is completely unknown; therefore, we assessed its role in pancreatic cancer. We found that C4orf47 was a direct target of HIF-1α and is upregulated in hypoxic conditions, in which it suppressed the cell cycle and inhibits cell proliferation through up-regulation of the cell cycle repressors Fbxw-7, P27, and p57; and the down-regulation of the cell cycle promoters c-myc, cyclinD1, and cyclinC. Furthermore, C4orf47 induced epithelial-mesenchymal transition and enhanced their cell plasticity and invasiveness. In addition, the p-Erk/p-p38 ratio was significantly enhanced and down-regulated CD44 expression by C4orf47 suppression, suggesting that C4orf47 is involved in pancreatic cancer dormancy under hypoxic conditions. Furthermore, the potential of C4orf47 expression was a good prognostic biomarker for pancreatic cancer. These results contribute to the elucidation of the pathology of refractory pancreatic cancer and the development of novel therapeutic strategies.

## Introduction

In the United States, approximately 60,430 new cases of pancreatic cancer are projected to be diagnosed in 2021 1. The incidence rate is increasing at 0.5% to 1.0% per year and pancreatic cancer is projected to be the second leading cause of cancer death in the United States by 2030 1,2. The majority (90%) of pancreatic neoplasms are pancreatic ductal adenocarcinomas (PDAC) with other subtypes including adenocarcinomas, pancreatoblastomas, and neuroendocrine tumors. The refractory state may be due to the difficulty of early diagnosis, which makes radical surgery difficult in many cases, and the absence of effective long-term therapeutic agents 3-5. Therefore, analysis of pancreatic cancer pathogenesis and the development of effective new therapies are urgent issues.

A characteristic feature of PDAC is its highly hypoxic state 6-10. Recently, hypoxic environments have attracted attention as a component of the cancer microenvironments. Normally, our tissues are in a state of 1% O_2_, while cancer tissues are in a state of 0.1% O_2_, which is an extremely hypoxic condition 11-14. Therefore, it is possible that various molecules and signaling systems are up-regulated or activated under hypoxic conditions that create a microenvironment that contributes to survival and induction of malignant traits but is unfavorable to cells.

Previously, we have shown that the expressions of leucocyte antigen related protein tyrosine phosphatase-interacting protein (liprin)-α4, master-mind like 3 (MAML3), recombination signal binding protein for immunoglobulin kappa J region (RBPL) and family with sequence similarity 115, member C (FAM115C) increased under hypoxic conditions in pancreatic cancer. Liprin-α4, MAML3 and RBPJ contributed to the proliferation, migration and invasion in pancreatic cancer 15,16. FAM115C was involved with the inhibition of invasiveness in pancreatic cancer and it could be a biomarker for good prognosis 17. Each molecule that was upregulated under hypoxia has its specific biological significance in pancreatic cancer.

Recently, we also identified the centrosome-associated protein chromosome 4 open reading frame 47 (C4orf47), which is upregulated in the hypoxic conditions observed in pancreatic cancer. C4orf47 is reported to be a centrosome-associated protein 18, however, there are few reports about the functional role of C4orf47. Especially, any reports of the biological significance for cancer have not been found. We thought that C4orf47 should play a pivotal role under hypoxia in pancreatic cancer throughout our previous research findings described above. Therefore, in this study, we analyzed the biological significance of C4orf47 in pancreatic cancer and characterized the cancer pathogenesis of PDAC to develop novel therapeutic strategies.

## Materials and methods

### Cell culture and reagents

Two human PDAC (ASPC-1 and SUIT-2) and normal (HPDE and 293FT) cell lines were used in this study. Each cell line was maintained in RPMI 1640 medium supplemented with 10% fetal calf serum (FCS; Life Technologies, Grand Island, NY) and 100 units/ml penicillin and 100 μg/ml streptomycin (Nacalai Tesque, Kyoto, Japan). To establish normoxic conditions, cells were cultured in 5% CO_2_ and 95% air. To establish hypoxic conditions, cells were cultured in 1% O_2_, 5% CO_2_ and 94% N_2_ in a multi-gas incubator (Sanyo, Tokyo, Japan).

### RNA interference

C4orf47 (ON-TARGETplus^TM^ SMART pool, No.L-033933), HIF-1α (ON-TARGETplus^TM^ SMART pool, No. L-004018), and negative control siRNA (ON-TARGETplus™ Control non-targeting siRNA, No. D-001810) were purchased from Dharmacon (Lafayette, CO). Transfection was carried out at 37°C for 48 h according to the manufacturers protocol; cells (2.0 × 10^5^ cells/well) were seeded in 6-well plates and transfected with 20 nM siRNA under normoxia using Lipofectamine RNAiMAX reagent (Invitrogen; Thermo Fisher Scientific, Inc.).

### Western blotting analysis

Western blotting was performed as described previously 19. The protein-transferred membranes were incubated for 1 h at room temperature and then overnight at 4 °C with primary antibodies against C4orf47 (1:500, No. APR69924_P050; Aviva System Biology), CyclinC (1:1000, No. ab85927; Abcam), CyclinD1 (1:200, No. sc-246; Santa Cruz Biotechnology, Inc.), CDK4 (1:200, No. H-22; Santa Cruz Biotechnology, Inc.), Fbxw-7 (1:1000, No. ab109617; Abcam), c-Myc (1:200, No. sc-40; Santa Cruz Biotechnology, Inc.), Erk1/2 (1:1000, No.9102; Cell Signaling Technology), p-Erk1/2 (1:1000, No.9101; Cell Signaling Technology), p-p38 (1:100, No. sc-166182; Santa Cruz Biotechnology, Inc.), MEK1/2 (1:200, No. sc-81504; Santa Cruz Biotechnology, Inc.), p-MEK1/2 (1:1000, No. sc-7995; Santa Cruz Biotechnology, Inc.), Akt1/2 (1:1000, No. sc-8312; Santa Cruz Biotechnology, Inc.), p-Akt1/2/3 (1:100, No. sc-514032; Santa Cruz Biotechnology, Inc.), e-cadherin (1:200, No. sc-8426; Santa Cruz Biotechnology, Inc.), vimentin (1:1,000, No. sc-6260; Santa Cruz Biotechnology, Inc.), Twist (1:200, No. sc-81414; Santa Cruz Biotechnology, Inc.), Snail-1 (1:200, No. sc-271977; Santa Cruz Biotechnology, Inc.), or Slug (1:200; No. sc-166476; Santa Cruz Biotechnology, Inc.). The membranes were then incubated for at least 3 h at room temperature with horseradish peroxidase-linked anti-mouse antibody (1:10,000, No. NA931; Amersham Biosciences; Cytiva), horseradish peroxidase-linked anti-rabbit antibody (1:10,000, No. NA934; Amersham Biosciences; Cytiva), or horseradish peroxidase-linked anti-goat antibody (1:10,000, No. sc-2020; Santa Cruz Biotechnology, Inc.). Immunocomplexes were detected with Amersham ECL Prime Western Blotting Detection Reagent (GE Healthcare, Tokyo, Japan) and visualized with EZ Capture ST (ATTO, Tokyo, Japan). We used α-Tubulin (1:1000, Sigma-Aldrich, St. Louis, MO, USA) as a protein loading control. Results were quantified using the Image J software program (The National Institutes of Health, USA).

### PCR amplification and cloning of the C4orf47 promoter

Genomic DNA was isolated from the SUIT-2 cell line using the Monofus DNA Kit IV (GL Science, Japan) and quantified. The *C4orf47* promoter sequence and *HRE* (*Hypoxia Response Element*) were amplified from the genomic DNA by PCR. Construct ① (-267 bp to 761 bp w.r.t. TSS + 1) used a primer pair that included the KpnI and NheI sites (forward, 5'-ATTGCCACCGCAT ACGGAGAGTCAAGGGGGAG-3'; Reverse, 5'-ATTGCTAGC GGCCTTCTTGGACGCAAATG-3'), while construct ② (133bp ~ + 767bp w.r.t. TSS + 1) used a primer that included the KpnI and NheI sites (forward, 5'-ATTGCCACCGCATGCTTTTCTCTATGGACGAATAGTGG-3'; reverse, 5'-ATTGCTAGCCGTCCAAGAAGGCCATCCAT-3'), respectively. Construct ① was prepared to include both the *C4orf47* promoter sequence and *HRE*, and construct ② was prepared to include only the HRE. The PCRs were performed using KOD One PCR Master Mix (TOYOBO, Japan). The amplified products were subjected to agarose gel electrophoresis and the amplified DNA was excised from the gel, purified, cloned into pGL3-BasicVector (Promega, USA), and named pGL3 (C4orf47-①) or pGL3 (C4orf47-②). The constructs were introduced into DH5α cells, which were plated and cultured. Positive colonies were selected and the sequence of the cloned vector was confirmed by sequencing on an Applied Bio- systems 3130xl Genetic Analyzer (Applied Biosystems) using BigDye® Terminator v3.1.

### Dual luciferase assay

For the luciferase assay, 293 T cells were plated in 6-well plates and cultured overnight. Next, reporter gene constructs pGL3 (C4orf47-①), pGL3 (C4orf47-②), or pGL3-empty vector were introduced into the cells with phRV-SV40 using the Lipofectamine 3000 reagent. After 48 h, the cells were incubated under normal oxygen and hypoxic conditions for an additional 24 h. Luciferase activity was analyzed using the Dual Luciferase Assay Kit (Promega) following the manufacturer's protocol.

### Cell invasion assay

The motility of the pancreatic cancer cell lines was assessed by Matrigel invasion assay as previously described 20. Briefly, siRNA-transfected cells (2.0×10^5^) were placed in the upper chamber of a Transwell chamber and incubated for 18 h at normoxic conditions. The cells that invaded the lower chamber were fixed and stained with Diff-Quik reagents following the manufacturer's protocol (Sysmex Corporation). The stained cells were counted at an ×200 magnification under a light microscope (Nikon Eclipse TE 300; Nikon Corporation).

### Cell proliferation assay

Pancreatic cancer cell lines were transfected with C4orf47 siRNA or negative control siRNA and seeded onto 96-well plates (4.0×10^3^ cells/well). These cells were then incubated at 37 °C for 0, 24, 48 or 72 h. In cytotoxic assays, siRNA-transfected cells were incubated with or without 0-50 nM Paclitaxel (PTX) at 37 °C for 48 h. Cell Count Reagent SF (Nacalai Tesque, Inc.) was then added to the cells and incubated at 37 °C for 1 h. Cell proliferation was assessed by absorbance at 492 nm using a plate reader (Biotrak visible plate reader; Amersham Biosciences; Cytiva) with a 620-nm reference wavelength.

### *In vivo* xenograft tumor model

Four-week-old female thymus-free nude mice (BALB/c nu/nu) were purchased from the Charles River Laboratories of Japan and acclimated for 1 w. All experimental procedures were approved by the Animal Welfare Committee of Kyushu University (Permit No. A21-3437-1) and were conducted in accordance with the “Guidelines for the Proper Conduct of Animal Experiments” (Science Council of Japan). Briefly, all mice were housed and maintained in the specific pathogen-free animal facility at Kyushu University under 12-h housing conditions of 26-28°C temperature, 40-70% humidity, and 8:00 am to 8:00 pm light/dark cycle. Food and water were provided freely. Every effort was made to minimize the number of animals used and their suffering. The humane endpoint of euthanasia was defined at a tumor diameter greater than 10 mm or prolonged pain. Mice with skin ulcers at the tumor graft site were euthanized as an indication for the humane endpoint. For the humane and experimental endpoints, mice were euthanized by overdose of an inhaled sevoflurane anesthetic, and the death of the mice was confirmed by cardiopulmonary arrest and loss of pupillary light reflex. *C4orf47* siRNA or control siRNA-transfected SUIT-2 cells were subcutaneously implanted in both inguinal regions of the nude mice (1.0 × 10^6^ cells in Matrigel per animal; n = 4 per group). Tumor size was measured every 2 d and tumor volume was calculated as follows: L×D^2^, where L is the longest diameter and D is the smaller of the two orthogonal diameters of the tumor.

### Cell cycle analysis

Cells (2.0×10^5^ cells/well) were treated for 48 h with *C4orf47* siRNA or negative control siRNA. Cells were harvested by trypsinization and fixed in ice-cold 70% ethanol for at least 1 h. Cell pellets were incubated and stained for 30 min at room temperature in 1 ml PBS containing 50 µg propidium iodide (Sigma-Aldrich; Merck KGaA), 0.1% Triton X-100, 1 mM/l EDTA, and 0.5 mg ribonuclease A (Sigma-Aldrich; Merck KGaA). After staining, samples were analyzed using FACScan (BD Biosciences) and BD CellQuest™ Pro software 6.0 (BD Biosciences) at 20,000 events per sample. The percentages of cells in G0/G1 phases were calculated for each sample.

### Immunohistochemistry

Tissue samples obtained from mice transplanted with SUIT-2 cells transfected with *C4orf47* siRNA or negative control siRNA were used for analysis. Immunohistochemical staining was performed on paraffin-embedded tissue sections with either c-myc (1:50; product code Sc-246, Santa Cruz Animal Health), CyclinD1 (1:50; product code Sc-40, Santa Cruz Animal Health). Endogenous peroxidase activity was blocked for 5 min using 3% hydrogen peroxide solution. Antigen activation was performed using a high-pressure method with Target Retrieval Solution, pH 9.0 (Agilent Technologies, Inc.) for 10 min. Slides were incubated with primary antibodies overnight at 4 °C, and then incubated with Histofine Simple Stain MAX-PO (M) (4 µg/ml; goat. No. 424131) or Histofine Simple Stain MAX-PO (R) (4 µg/ml; cat. No. 424131; Nichirei Biosciences, Inc.) for 40 min at room temperature. Labeled proteins were visualized using diaminobenzidine (DAB). Counterstaining was performed with hematoxylin for 3 min at room temperature. C-myc and CyclinD1 positive cells were observed at 400x magnification.

In the analysis of c-myc expression and cyclinD1 expression of mouse xenograft tumors, three different preparations were stained and we could observe the c-myc expression and cyclinD1 expression in all specimen. Negative controls were obtained by omitting the first antibodies 21. For the assessment of the expression, we selected five microscopic fields at 400× magnification and positively stained areas were quantified using Image J.

### Bioinformatics analysis

The published overall survival data set (including published human data) is available from The Cancer Genome Atlas-Pancreatic Adenocarcinoma (TCGA-PAAD) through The Human Protein Atlas (https://www.proteinatlas.org).

### Statistical analysis

All data are expressed as the mean ± standard deviation (SD). The unpaired Student's t-test was used for the comparison of mean values between two groups. Calculations were conducted using JMP 14.0 software (SAS Institute) or Microsoft Excel software (Microsoft). P-values of < 0.05 was considered to be statistically significant.

## Results

### C4orf47 is a direct target gene of HIF-1α

**Figure 1:**
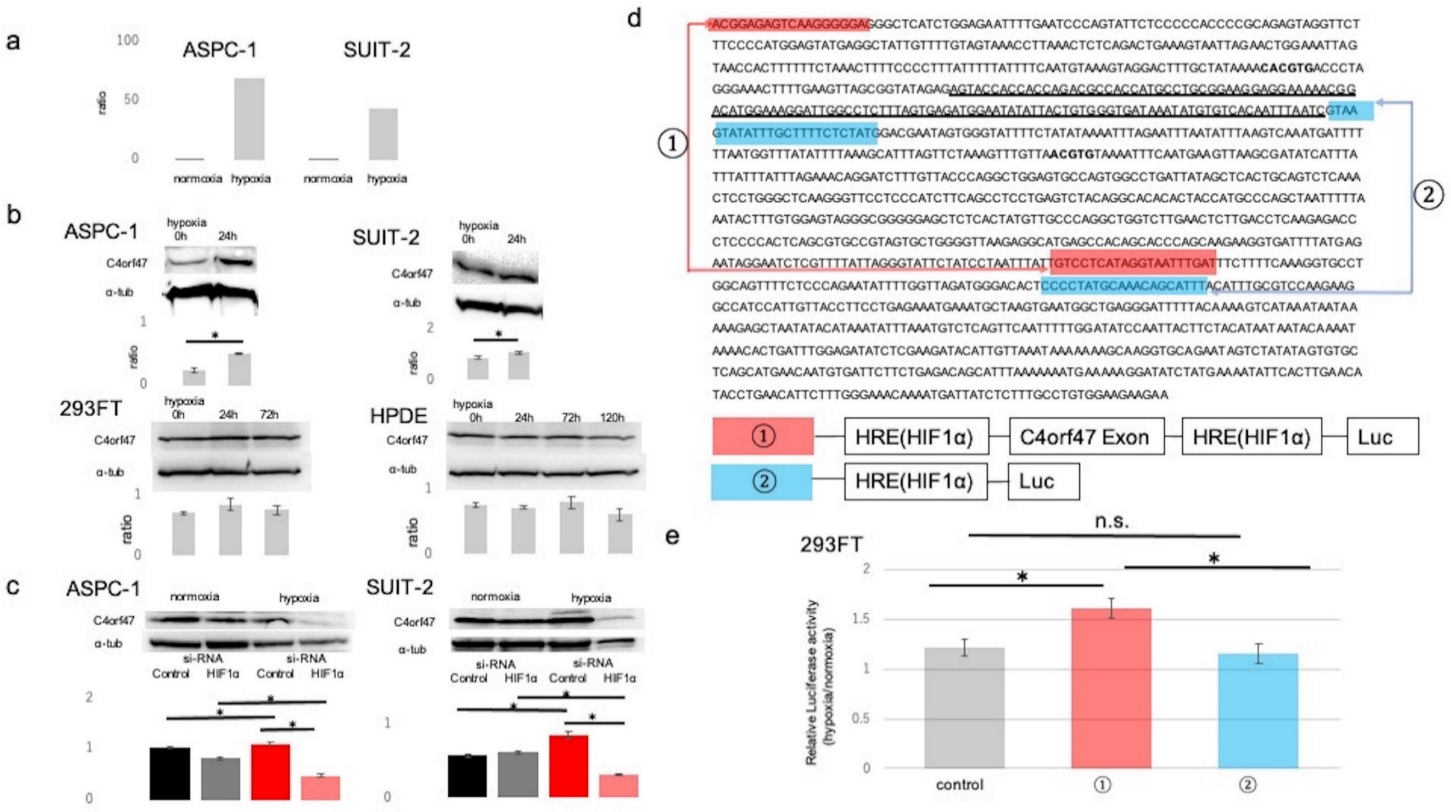
** C4orf47 is a direct target gene of HIF-1α. (a)** Microarray analysis was performed pancreatic cancer cell lines cultured in 20% O_2_ and 1% O_2_. **(b)** Western blotting analysis of C4orf47 expression in PDAC, 293FT, and normal pancreatic duct cell lines in hypoxic (1% O_2_) conditions after 0, 24, 72, and 120 h. Relative values of quantified protein expression are shown. **(c)** Cells transfected with negative control siRNA and HIF-1α siRNA were incubated under normal oxygen for 48 h and then divided into normal oxygen and hypoxic environments for and additional 24 h. Western blotting analysis of C4orf47 expression in PDAC cell lines and relative values of quantified protein expression are shown. **(d)** The transcription start site (TSS) of the *C4orf47* gene was identified with the Ensembl Genome Browser. The sequence of *C4orf47* from Human GRCh38/hg38 was obtained using the UCSC Genome Browser. The gene sequence around the TSS matched the *C4orf47* sequence from Human GRCh38/hg38 in ApE (A plasmid editor). The TSS is underlined and HRE is in bold font. Construct ① was created with both the *C4orf47* promoter sequence and HRE, while construct ② contains only the HRE. Construct ① is indicated by red arrows and construct ② is indicated by blue arrows. Plasmid vectors incorporating luciferase at the ends of constructs ① and ② were also designed. **(e)** 293FT cells transfected with constructs ① and ② and the pGL3-empty vector were cultured under normoxia for 48 h, divided into normoxic and hypoxic conditions, and cultured for another 24 h. Luciferase expression was measured in triplicate-wells. The ratio of luciferase activity in 293FT cells cultured under hypoxia to those cultured under normal oxygen was calculated. Data are shown as mean ± standard deviation. *p<0.05. n.s., no significant difference.

First, we performed microarray analysis of pancreatic cancer cell lines cultured in normal (20% O_2_) and hypoxic (1% O_2_) environments to identify molecules that are upregulated in hypoxic environments in pancreatic cancer. The results showed that *C4orf47* was upregulated approximately 68-fold in ASPC-1 and 42-fold in SUIT-2 cells cultured in hypoxia (Fig. 1a). The C4orf47 protein expression analysis showed that C4orf47 was significantly enhanced in both ASPC-1 and SUIT-2 cell lines cultured under hypoxia for 24 h when compared with the normal 293FT and HPDE cell lines, which had similar C4orf47 under both normoxia and hypoxia (Fig. 1b). This suggests that C4orf47 protein expression was significantly upregulated under hypoxia in ASPC-1 and SUIT-2, consistent with the microarray results.

**Figure 2:**
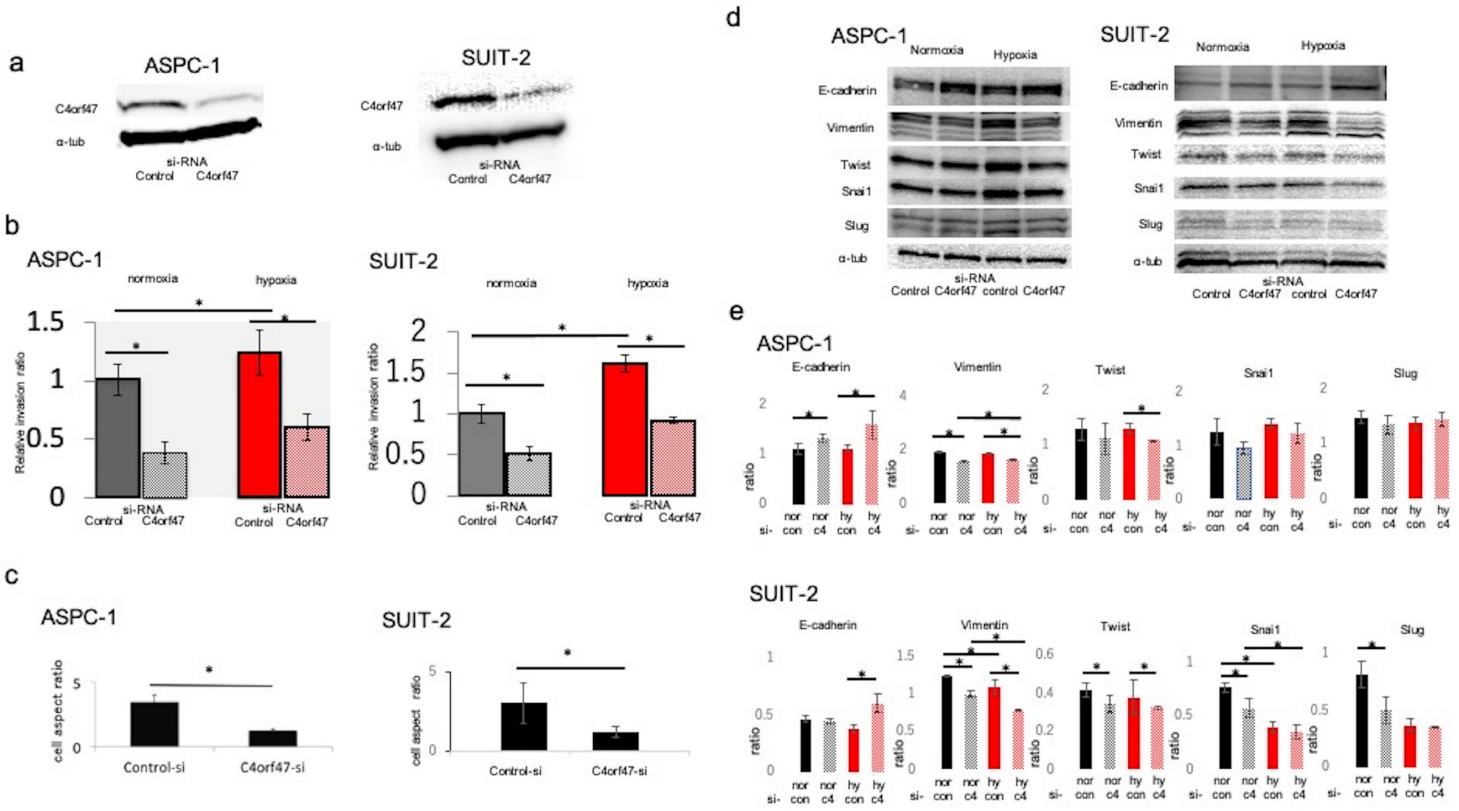
** C4orf47 is involved in enhanced invasion via epithelial-mesenchymal transition (EMT) in pancreatic cancer cell lines.** The PDAC cell lines were cultured under normal oxygen for 48 h after transfection with negative control or *C4orf47* siRNA, and then divided into normal oxygen and hypoxic environments for 24 h. **(a)** Western blotting analysis of C4orf47 expression; **(b)** Matrigel invasion assay; and **(c)** optical microscopy (Nikon Eclipse TE 300; Nikon Corporation) under 200x and the aspect ratio of cells were calculated.** (d)** Western blotting analysis and **(e)** the quantification of E-cadherin, Vimentin, Twist, Snail1 and Slug relative expression during EMT. Data are shown as mean ± standard deviation. *p<0.05.

Since *C4orf47* is upregulated under hypoxia, we evaluated its association with Hypoxia Inducible Factor-1α (HIF-1α), a transcription factor that is important in hypoxic conditions. Suppression of HIF-1α resulted in no change in expression of *C4orf47* under normoxia but significantly decreased *C4orf47* expression under hypoxia in the ASPC-1 cell line. Similarly, in the SUIT-2 cell line, no change was observed in *C4orf47* expression under normoxia, while its expression was significantly decreased by HIF-1α suppression under hypoxia (Fig. 1c). Based on these results, we hypothesized that C4orf47 may be associated with the expression of HIF1α. Therefore, we identified a region containing the exon of the *C4orf47* promoter (Fig. 1d: underlined sequence) and a hypoxia responsive element (HRE; Fig. 1d: bold sequence) that binds to HIF-1α 22,23. We engineered a construct of the HRE with (Fig. 1d, ①) or without (Fig. 1d, ②) the exon of the *C4orf47* promoter.

Plasmid vectors incorporating luciferase at both ends of each construct were created and transfected into 293FT cells. The cells transfected with the empty vector were designated as control cells. The 293FT cells transfected with each vector were cultured in normoxic (20% O_2_) and hypoxic (1% O_2_) environments, and the ratio of luciferase activity in 293FT cells cultured in the hypoxic environment to that in the normoxic environment was calculated. The ratio was significantly higher in 293FT cells transfected with construct ①containing HRE with the exon of *C4orf47* promotor than control cells (Fig. 1e). Moreover, there was no significant difference between the control cells and cells transfected with construct ②. Thus, the cells transfected with construct ① showed significantly increased luciferase activity due to the action of HIF-1α in a hypoxic environment, suggesting that *C4orf47* is the direct gene target of HIF-1α.

### C4orf47 enhances cell invasion via epithelial-mesenchymal transition in pancreatic cancer cell lines

To analyze the biological significance of C4orf47 in pancreatic cancer, we first examined its involvement in cell invasive potential. The C4orf47 suppressor system was analyzed by using siRNA to suppress *C4orf47* mRNA expression. We confirmed that *C4orf47* in ASPC-1 and SUIT-2 was sufficiently suppressed when the cells were transfected with si-C4orf47 (Fig. 2a). Because *C4orf47* expression was enhanced by HIF-1α under hypoxic conditions, we used cells cultured under hypoxic conditions as a positive control and evaluated cell infiltration capacity using Matrigel infiltration test. The invasive ability of the ASPC-1 cells was significantly enhanced in the control group in hypoxia, but was significantly decreased when *C4orf47* was suppressed in both normoxia and hypoxia (Fig. 2b). In the SUIT-2 cells, the invasive ability was significantly enhanced in the control group under hypoxic conditions and significantly decreased when *C4orf47* was suppressed in both normoxic and hypoxic conditions (Fig. 2b). These results suggested that C4orf47 helps enhance the invasive potential of pancreatic cancer cells.

In the ASPC-1 and SUIT-2 cell lines, C4orf47 suppression decreased the cell length-to-diameter ratio, and the cells became morphologically spindle-shaped (Fig. 2c), which suggested that suppression of *C4orf47* resulted in mesenchymal epithelial conversion 24-26. Therefore, we evaluated markers of epithelial-mesenchymal conversion using western blotting analysis. In the ASPC-1 cell line, E-cadherin was upregulated while Vimentin was down-regulated in cells with suppressed *C4orf47* expression grown under both normoxia and hypoxia. Twist was down-regulated by *C4orf47* suppression in hypoxia, while Snail and Slug showed no significant change in their expression in response to hypoxia. Although the expression patterns for E-cadherin, Vimentin, and in response to C4orf47 suppression in SUIT-2 cells both normoxia and hypoxia were similar to that observed in ASPC-1 cells, Snail and Slug were down-regulated by the *C4orf47* suppression in SUIT-2 cells grown under normoxia (Fig. 2d,e). These results suggested that C4orf47 enhances the invasive potential of cells through an epithelial-mesenchymal transition.

### C4orf47 reduces proliferation and drug sensitivity in pancreatic cancer cell lines

**Figure 3:**
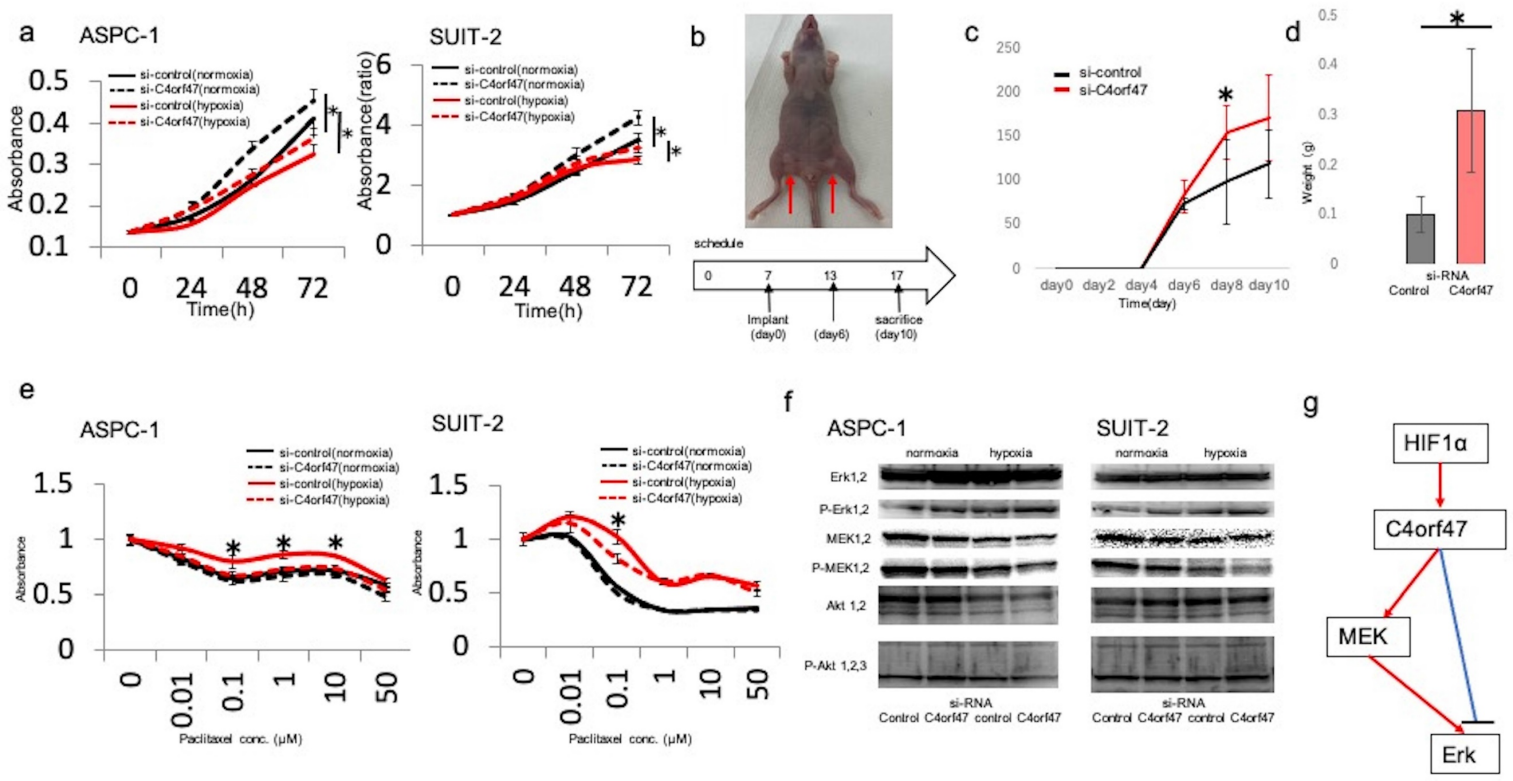
** C4orf47 is involved in the suppression of proliferation and drug sensitivity in pancreatic cancer cell lines. (a)** After negative control and *C4orf47* siRNA were introduced into PDAC cell lines, they were incubated under normal oxygen for 48 h and then divided into normal oxygen and hypoxic conditions. The proliferation assay was conducted at 0, 24, 48, and 72 h after normoxic and hypoxic incubation. **(b)** Four-week-old female thymus-free nude mice (BALB/c nu/nu) were acclimated for 1 w before Matrigel mixtures containing 1 × 10^6^ SUIT-2 cells transfected with negative control siRNA or C4orf47 siRNA were subcutaneously implanted in inguinal regions of the hind legs (arrows) (n=4 per group).** (c)** Tumor size was measured every 2 d and tumor volume was calculated after collection 10 d after skin grafting. **(d)** Tumors were removed from euthanized mice and weighed. **(e)** Cells transfected with control or *C4orf47* siRNA were cultured with 0-50 µM PTX under normoxia and hypoxia for 48 h before WST-8 was added and cultured for another 1 h at 37°C and measured using spectrophotometer. The absorbance of surviving cancer cells was significantly different between negative control siRNA and C4orf47 siRNA under hypoxia. * indicates p<0.05. **(f)** PDAC cells were transfected with negative control or C4orf47 siRNA and then cultured under normoxia for 48 h. The cells were divided into normoxia and hypoxia groups and cultured for another 24 h. Expression of MEK 1/2, p-MEK 1/2, Erk 1/2, p-Erk 1/2, Akt 1/2, p-Akt 1/2/3 were analyzed by western blotting. **(g)** Predicted downstream of C4orf47. red line: up-regulation, blue line: down-regulation.

Next, we asked if C4orf47 could affect the proliferative capacity in pancreatic cancer cells. The ASPC-1 cell line showed decreased proliferation under hypoxic conditions, whereas *C4orf47* suppression significantly increased proliferation under both normoxia and hypoxia. Similarly, in the SUIT-2 cell line, cell proliferation decreased in a hypoxic environment and significantly increased under both normoxia and hypoxia when *C4orf47* was suppressed (Fig. 3a).

To verify whether the *in vitro* results were reflected *in vivo*, we examined the proliferative ability of the pancreatic cells expressing siC4orf47- and si-control-transfected SUIT-2 cell lines after subcutaneous injection in nude mice (Fig. 3b). Both cell lines showed viability on day 6 after injection, and the *C4orf47*-suppressed pancreatic cancer cell lines developed significantly larger tumors than the si-control-transfected pancreatic cancer cell lines on day 8 after injection (Fig. 3c). The tumors of the pancreatic cancer cell lines transfected with si-C4orf47 tended to be larger than those that developed from the si-control-transfected cells at day 10 after injection, but the difference was not significant (Fig. 3c). The control pancreatic cancer cell specimens weighed 0.39 g, 0.22 g, 0.19 g, and 0.44 g; while and the si-control-transfected pancreatic cancer cell specimens weighed 0.13 g, 0.11 g, 0.11 g, and 0.05 g, indicating that the si-C4orf47-repressed pancreatic cancer cell derived tumors were significantly heavier (Fig. 3d). These results suggested that inhibition of *C4orf47* enhanced the proliferative potential of pancreatic cancer cells *in vivo*.

Next, we evaluated the drug sensitivity of pancreatic cancer cell lines transfected with si- C4orf47 or control constructs. Paclitaxel (PTX) is an inhibitor of microtubule depolymerization, and because C4orf47 is involved in centromere function 18, we hypothesized that C4orf47 may affect PTX sensitivity. Drug sensitivity was reduced in the hypoxic environment in the ASPC-1 cell line when compared to normoxic conditions. Although *C4orf47* suppression showed no significant difference in the number of viable cancer cells at any drug concentration under normoxia, under hypoxia, *C4orf47* suppression significantly reduced the number of viable cancer cells and increased drug sensitivity (Fig. 3e). In the SUIT-2 cell line, si-control cells grown under hypoxia had reduced drug sensitivity compared with normoxic conditions, in which there was no significant difference in the number of viable cancer cells between drug dosages. However, in cells with suppressed *C4orf47*, the number of viable cancer cells was significantly lower at 0.1 μM PDX under hypoxia when compared with normoxia, indicating *C4orf47* suppression increased sensitivity to PDX in hypoxic environments (Fig. 3e). Together, these results suggested that C4orf47 is involved in the suppression of cell proliferation and drug sensitivity under hypoxia in pancreatic cancer cells.

Next, we evaluated the downstream target of C4orf47. The molecules of MAPK and PI3k that contribute to the proliferation and invasiveness were estimated. When C4orf47 was inhibited, the expression of the phosphorylated ERK (pERK) increased and the phosphorylated MEK (pMEK) inversely decreased both under normoxia and hypoxia (Fig. 3f). There was no difference of the phosphorylated AKT. These results suggest that the up-stream target of C4orf47 is HIF-1α and the down-stream target of C4orf47 is pERK (Fig. 3g). pMEK may be affected by the the negative feedback.

### C4orf47 is involved in cell dormancy

**Figure 4:**
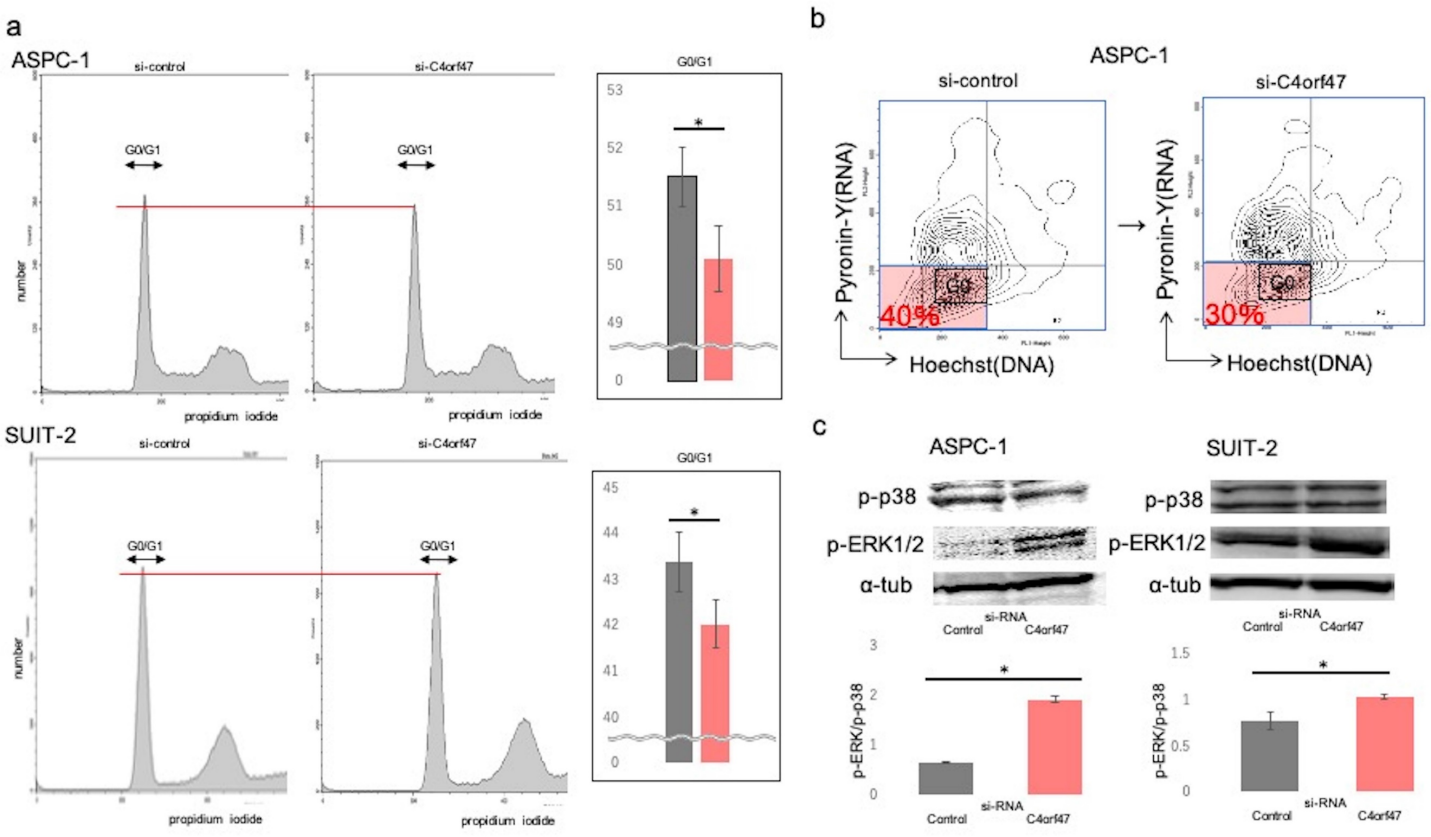
** C4orf47 is involved in G0/G1 arrest. (a)** 2 × 10^5^ (cells/well) PDAC cells were incubated under normal oxygen for 48 h after transfection with negative control or *C4orf47* siRNA and then collected by trypsin treatment and fixed in ice-cold 70% ethanol. Cell pellets were stained with propidium iodide and analyzed at 20,000 events per sample using flowcytometry. For each sample, the percentage of cells in G0/G1 phase was calculated. **(b)** Cell pellets were stained with Pyronin Y and Hoechst Y for FACS analysis. The G0 fraction of Hoechst Y^low^, PyroninY^low^ cells is shown. **(c)** After PDAC cell lines were transfected with control and *C4orf47* siRNA, they were cultured under normal oxygen for 48 h and divided into normal oxygen and hypoxia treatment groups for 24 h. Western blotting analysis of P-p38 and p-ERK1/2 was performed and the P-p38 to p-ERK1/2 ratio was calculated. Data are shown as mean ± standard deviation. * indicates p<0.05.

To analyze the mechanism by which C4orf47 inhibits cell proliferation, we performed cell cycle analysis and found that *C4orf47* suppression significantly decreased G0/G1 in the ASPC-1 and SUIT-2 cell lines (Fig. 4a). Pyronin-Y-Hoechst staining was used to evaluate G0/G1^27^ and confirmed that the G0 fraction was reduced in ASPC-1 cells with suppressed *C4orf47* expression (Fig. 4b).

Our previous results showed that C4orf47 suppressed cell proliferation and enhanced invasion. Moreover, it was reported that the suppressed proliferation and enhanced invasion phenotypes are associated with cell dormancy 28-32. Therefore, we next determined the p-Erk/p-p38 ratio, which is an indicator of dormancy 33-36. A high p-Erk/p-p38 ratio indicates active proliferation and low for dormancy. We found that *C4orf47* suppression significantly increased the p-Erk/p-p38 ratio in both ASPC-1 and SUIT-2 cell lines (Fig. 4c), suggesting C4orf47 is involved in regulating cell dormancy.

**Figure 5:**
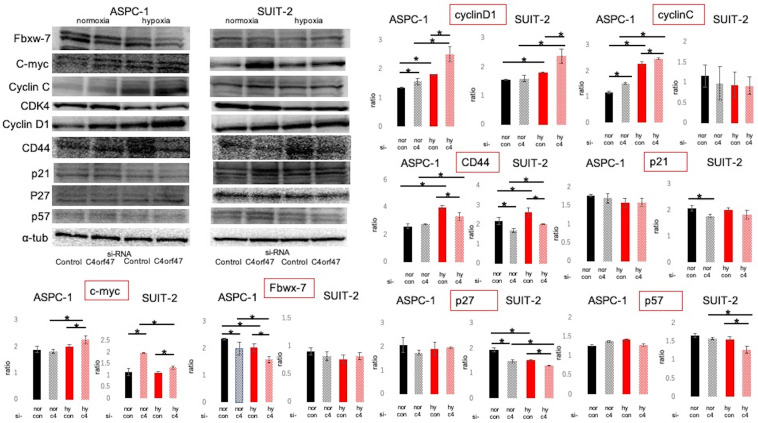
** Evaluation of cell cycle-related proteins expressed.** PDAC cells were transfected with negative control or C4orf47 siRNA and then cultured under normoxia for 48 h. The cells were divided into normoxia and hypoxia groups and cultured for another 24 h. Expression of Fbxw-7, c-myc, CyclinC, CDK4, CyclinD1 cancer stem cell markers and p21, p27, and p57 cell cycle suppressors were analyzed by western blotting. The relative values of quantified protein expression are shown as mean ± standard deviation. * indicates p<0.05.

**Figure 6:**
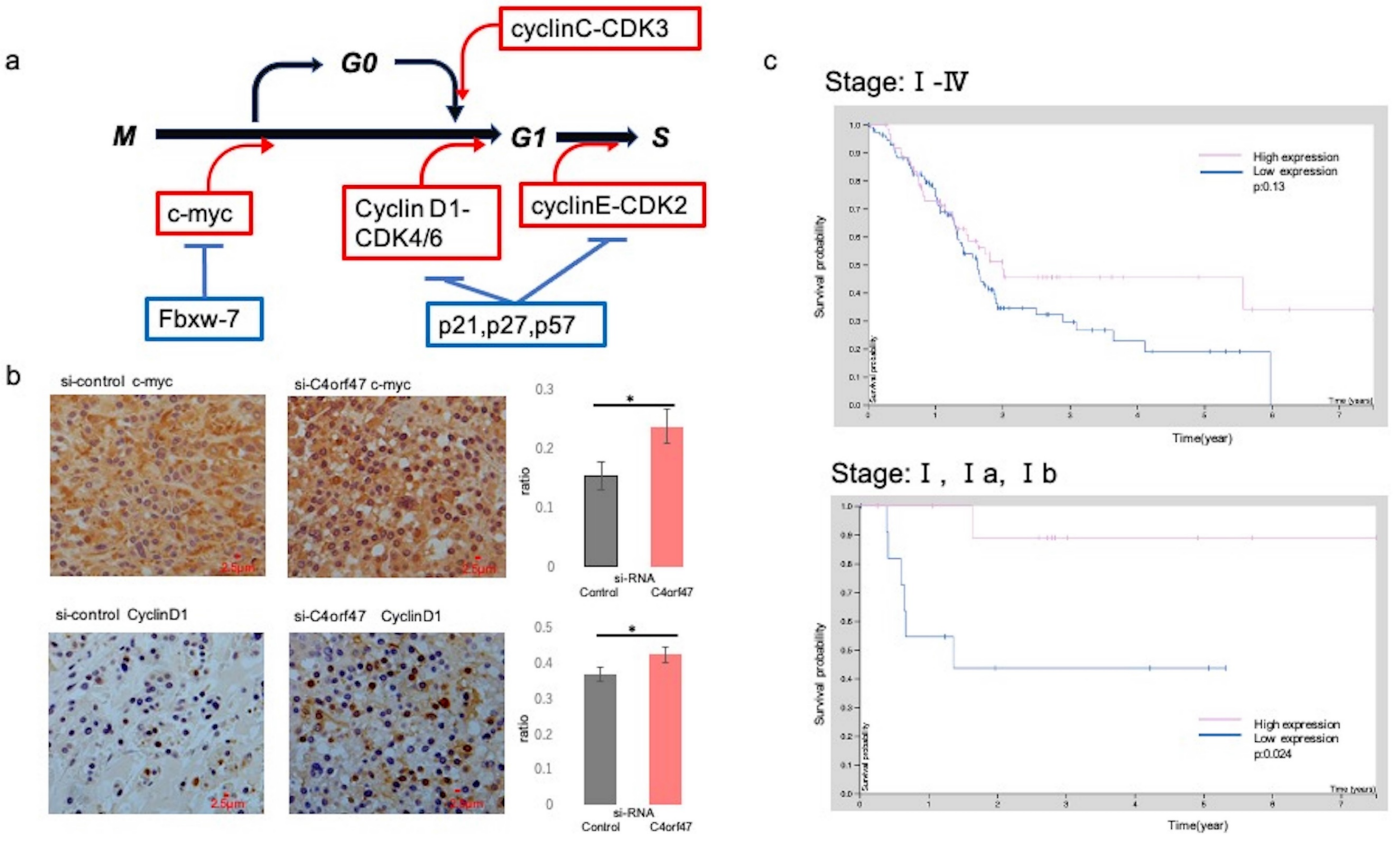
** C4orf47 expression is a potential biomarker for a favorable prognosis for pancreatic cancer. (a)** Schematic of the cell cycle examined in this study.** (b)** Immunohistology images of c-myc and CyclinD1 in xenograft tumors of SUIT-2 cells transfected with negative control and *C4orf47* siRNA. Graphs quantifying the relative value of positively stained area are shown on the right. **(c)** The Cancer Genome Atlas-Pancreatic Adenocarcinoma (TCGA-PAAD) available from The Human Protein Atlas and the overall survival curves obtained.

Cell cycle-associated proteins were subsequently evaluated by Western blotting (Fig. 5) 37-47. The roles of the evaluated molecules in the cell cycle are shown in Figure 6a. C- myc and CyclinD1 expression was significantly upregulated in hypoxic conditions in both ASPC-1 and SUIT-2 cell lines upon C4orf47 suppression. CD44 48, a marker of cancer stemness, was upregulated in the hypoxic control group and downregulated in the C4orf47- suppressed group compared with normoxia. The expression of p21, p27, and p57, known cell cycle repressors, was unchanged in the ASPC-1 cell line in the C4orf47 repressor group, while p27 and p57 were significantly down-regulated in the SUIT-2 cell line under hypoxic conditions. The expression of Fbxw-7, which is involved in the degradation of c-myc, was significantly reduced by suppressing C4orf47 in the ASPC-1 cell line, but not in the SUIT-2 cell line. CyclinC 42, which forms a complex with CDK3 and phosphorylates Rb proteins to promote escape from G0 phase, was upregulated in the ASPC-1 cell line upon C4orf47 suppression, but was unchanged in the SUIT-2 cell line.

*In vitro*, c-myc and CyclinD1 were up-regulated in the *C4orf47*-suppressed cells under hypoxia in both ASPC-1 and SUIT-2 cell lines when compared with the siRNA-control cell groups. We then asked if these results could be validated *in vivo* by evaluating tissue specimens obtained from mice that received subcutaneous injections in Fig. 3b-d. The control group was characterized by the absence of or low expression of c-myc, whereas high c-myc expression was observed in the samples from the *C4orf47*-suppressed group. CyclinD1 was up-regulated in the *C4orf47*-suppressed group but not in the control group (Fig. 6b), further indicating that *C4orf47* suppression increased the expression of CyclinD1. These results suggested that C4orf47 suppresses cell proliferation by inducing G0/G1 arrest through the up-regulation of the cell cycle repressors Fbxw-7, P27, and p57; and the down-regulation of the cell cycle promoters c-myc, cyclinD1 and cyclinC (Fig. 6b). C4orf47 is transformed into Cancer Stem Cells (CSCs) like because it enhances CD44 expression and maintained a low p- Erk/p-p38 ratio, suggesting that it is involved in dormancy.

### C4orf47 expression could be a biomarker for favorable pancreatic cancer prognosis

To test if C4orf47 expression has the potential to serve as a biomarker for pancreatic cancer prognosis, we evaluated the survival analysis for C4orf47 from The Human Protein Atlas using the investigation of *C4orf47* mRNA expression. The high expression group tended to have a better prognosis than the low expression group when the evaluation used all stages, but there was no significant difference (p=0.13). However, the high expression group had a significantly better prognosis than the low expression group when the analysis was restricted to Stage I (P=0.024; Fig. 6c). These results suggested that C4orf47 may have potential as a prognostic biomarker for pancreatic cancer.

## Discussion

In our comprehensive analysis of pancreatic cancer pathology in hypoxic environments, we found that C4orf47 is upregulated in hypoxic environments. C4orf47 is a centrosome-associated protein 18 that is conserved in most ciliated eukaryotic cells and is highly expressed in ciliary axons and ciliary tips of the bronchus, pharyngeal nares, testis, and oviducts in humans. Although there are no current reports that its expression is significantly related to disease pathologies, we found that C4orf47 is involved in regulating cell dormancy in pancreatic cancer. The hypothesis of tumor dormancy originated from the clinical finding that cancer recurrence occurs years or decades after surgical resection of the primary tumor and is classically defined as the cessation of primary tumor growth or metastatic dissemination 29. Tumor mass dormancy has been proposed as a model in which the increase in cancer cell number through cell proliferation is balanced by its decrease through cell death, such that tumor mass dormancy includes angiogenic dormancy and immune-mediated dormancy. Another new type of dormancy is cellular dormancy, in which cancer cells are in a quiescent state and is characterized by minimal proliferation, minimal death, and plasticity. In this study, C4orf47 was found to suppress proliferative capacity, and EMT was induced by the expression of C4orf47 in a hypoxic environment. Cellular phenotypic plasticity was also observed in the mediation of MET through C4orf47 activation and suppression 49-51. The up regulation of cancer stem cell markers was also observed, suggesting cellular dormancy. This may be due to the induction of G0/G1 arrest through the suppression of c-myc and cyclinD1 expression. Conversely, the upregulation of the cell cycle repressors p21, p27, and p57 43-47 was unclear and further studies of the expression pathways of these factors are required. Indeed, dormancy is classically defined as the cessation of tumor growth at the primary site or metastatic dissemination, but our *in vivo* experiments only assessed tumor growth potential. In order to evaluate dormancy, it will be necessary to evaluate metastatic potential. Therefore, C4orf47 forced expression and C4orf47 suppression groups using shRNA should be developed to create a cancer metastasis model for future experiments.

Because PTX is an anticancer drug that is widely administered in Japan to treat pancreatic cancer that cannot be curatively resected 52 and its mechanism of action is to inhibit microtubule depolymerization 53-55, we hypothesized that PTX might have some interaction with C4orf47 as it is a centrosome-associated protein. Our finding that PTX sensitivity was enhanced by C4orf47 inhibition may pave the way for new pancreatic cancer chemotherapy development, such as the combination of PTX and C4orf47 inhibitors. However, C4orf47 inhibition increased cell proliferation; therefore, targeting C4orf47 as a therapeutic strategy may not be a simple approach. Focusing on cell proliferation may also be a good strategy to enhance C4orf47 expression.

The decreased proliferative potential of pancreatic cancer associated with C4orf47 expression suggests that C4orf47 expression may be a good prognostic biomarker for Stage I pancreatic cancer, but conversely, the dormant state of the cancer cells may also result in differences in C4orf47 expression. The number of Stage I cases is small and further studies are needed to extend the observation period for the investigation of the involvement of C4orf47 in disease recurrence. Although C4orf47 may be involved in cellular dormancy in pancreatic cancer, its role in other carcinomas is completely unknown. In fact, analysis of C4orf47 expression and survival in other carcinomas has shown that the prognosis of lung cancer, breast cancer, and uterine cancer was significantly prolonged in the high C4orf47-expressing group (Supplementary Fig. a). However, glioma, thyroid cancer, colorectal cancer, liver cancer, kidney cancer, prostate cancer, testicular cancer, and melanoma (Supplementary Fig. b) showed significantly worse prognosis in the high C4orf47-expressing group. Thus, the analysis of the biological significance of C4orf47 in other carcinomas may be a useful direction for future research.

## Conclusion

**Figure 7:**
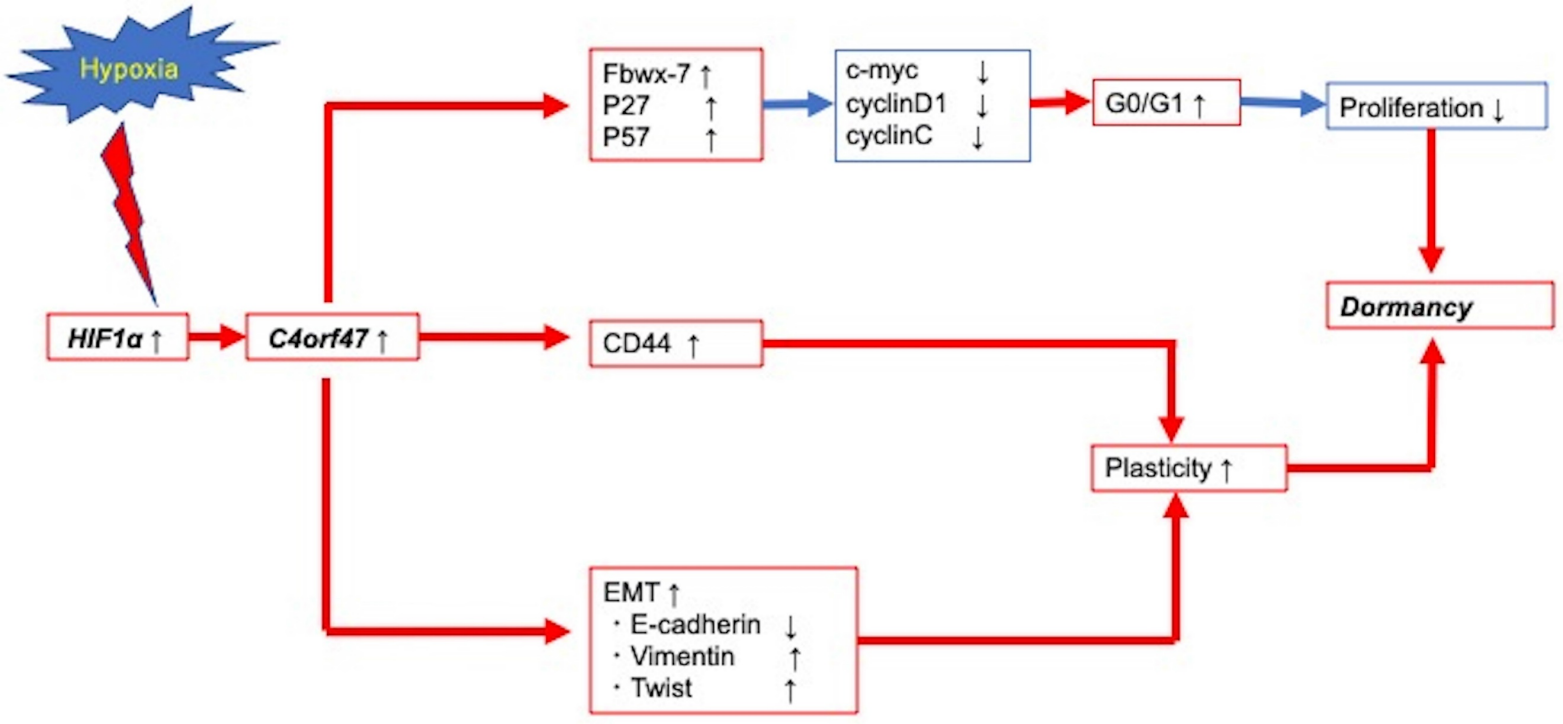
** Conclusion schema of this paper.** C4orf47 is involved in dormancy in hypoxia primarily through three transformational changes in the cell cycle, EMT, and CD44. red line: up-regulation, blue line: down-regulation.

Figure 7 presents a schematic of the findings from our research of the cellular and molecular mechanisms of C4orf47 in pancreatic cancer. First, *C4orf47* is a direct gene target of HIF-1α in pancreatic cancer cells and is up-regulated under hypoxic conditions. Second, C4orf47 suppresses cell proliferation by inducing G0/G1 arrest through the up-regulation of the cell cycle repressors Fbxw-7, p27, and p57 and the down-regulation of the cell cycle promoters c-myc, cyclinD1, and cyclinC. Third, C4orf47 induces cell plasticity and enhances the invasive potential by promoting EMT conversion. We also found that dormancy is a hallmark of cancer stem cells with respect to cancer recurrence, and the association of C4orf47 expression with CD44 expression may be involved in the maintenance of pancreatic cancer stem cells. These results suggest that C4orf47 is involved in pancreatic cancer cell dormancy under hypoxic conditions. Furthermore, we found that C4orf47 expression has potential as a prognostic biomarker for early stage pancreatic cancer. These results contribute to the elucidation of the cancer pathology of refractory pancreatic cancer and the development of novel therapies.

## Abbreviations

PDAC: pancreatic neoplasms are pancreatic ductal adenocarcinomas
liprin: leucocyte antigen related protein tyrosine phosphatase-interacting protein)
MAML3: master-mind like 3
RBPL: recombination signal binding protein for immunoglobulin kappa J region
FAM115C: family with sequence similarity 115, member C
C4orf47: chromosome 4 open reading frame 47
SD: standard deviation
HIF-1α: Hypoxia Inducible Factor-1α
HRE: hypoxia responsive element
PTX: Paclitaxel
